# Neuronal Dot1l Activity Acts as a Mitochondrial Gene-Repressor Associated with Human Brain Aging via H3K79 Hypermethylation

**DOI:** 10.3390/ijms24021387

**Published:** 2023-01-10

**Authors:** Hendrikus J. Van Heesbeen, Lars Von Oerthel, Paul M. De Vries, Cindy M. R. J. Wagemans, Marten P. Smidt

**Affiliations:** Swammerdam Institute for Life Sciences, University of Amsterdam, Science Park 904, Room C4.102, 1098 XH Amsterdam, The Netherlands

**Keywords:** epigenetics, gene regulation, dopamine, aging, respiratory genes, brain

## Abstract

Methylation of histone 3 at lysine 79 (H3K79) and its catalyst, a disrupter of telomeric silencing (DOT1l), have been coupled to multiple forms of stress, such as bioenergetic and ER challenges. However, studies on H3K79 methylation and Dot1l in the (aging) brain and neurons are limited. This, together with the increasing evidence of a dynamic neuroepigenome, made us wonder if H3K79 methylation and its activator Dot1l could play important roles in brain aging and associated disorders. In aged humans, we found strong and consistent global hypermethylation of H3K79 in neurons. Specific in dopaminergic neurons, we found a strong increase in H3K79 methylation in lipofucsin positive neurons, which are linked to pathology. In animals, where we conditionally removed Dot1l, we found a rapid loss of H3K79 methylation. As a consequence, we found some decrease in specific dopaminergic genes, and surprisingly, a clear up-regulation of almost all genes belonging to the family of the respiratory chain. These data, in relation to the observed increase in global H3K79 methylation, suggest that there is an inverse relationship between H3K79 methylation and the capacity of energy metabolism in neuronal systems.

## 1. Introduction

The dynamic character of the epigenome in post-mitotic neurons has gained much appreciation in the last decade, from being a mere debate, to evidence being gained for the complete and ongoing replacement of histone subunits in post-mitotic neurons, regardless of their epigenomic code [1,2]⁠⁠. Epigenomic profiles in neurons may partly reflect a steady-state rather than a long-lasting rigid code, per se. Such a dynamic balance of histone modification and eviction could shift in aging or diseased neurons, leading to global deregulation or facilitating the homeostatic adaptation of neurons, influencing healthy lifespan and disease progression [3]⁠⁠. In Parkinson’s disease (PD) especially, several mechanisms related to balanced histone acetylation and hydroxymethylation of DNA have been related to PD prevalence and disease mechanisms [4]⁠. However, related epigenetic mechanisms have been scarcely investigated, and many have not been investigated at all. Here, we have focused on the methylation of lysine 79 on histone 3 (H3K79), which is exclusively targeted by Dot1l in mammalian cells [5]⁠. Dot1l is recruited towards (active) genes via direct binding to Polymerase II and towards acetylated histones through recruitment by the transcription factor Af9 [6,7]⁠. The levels of Dot1l expression and recruitment have been related to an increase in methylation valence of H3K79 [8,9,10]⁠, generally leading to the accumulation of H3K79 dimethylation (H3K79me2). In an array of tested cell lines, H3K79me2 was found ubiquitously present, whereas H3K79me3 was limited [11]⁠.

Dot1l/DOT1l has been coupled to Wnt signaling via binding to beta-catenin-containing complexes [12,13]⁠. However, during the neuronal maturation phase in the mouse cortex, Dot1l is strongly down-regulated [14]⁠. In addition, several studies have coupled Dot1l chromatin regulation to Sirt1 and other Sir(t) proteins, linking Dot1(l) mechanistically to key factors of aging and inflammatory pathways [15,16,17].⁠

In cellular models, basal DOT1l levels and H3K79 methylation increase following cellular stress, such as responses to viral infections, by the manipulation of bioenergetics [18,19,20]⁠, or by matrix hyaluronan, which depletion reduces DA cell loss and α-synuclein in a mouse model of synucleinopathy [21,22]⁠. More recently, researching early life stress mouse models, both Dot1l and H3K79 methylation have been found to be long-lasting deregulated in medial spinal neurons [23]⁠. Finally, in neural stem cells Dot1l has been related to the ER stress response as well [24]⁠⁠. Taken together, DOT1l and H3K79me2 levels generally increase following a broad range of cellular stressors.

Among the diverse neuronal types, the DA neurons located in the substantia nigra pars compacta (SNc) are the most vulnerable to degenerate in PD, containing numerous arborizations that require structural and bioenergetical support [25,26,27]⁠. Even though, mechanistically and physiologically, Dot1l and H3K79 methylation relate to key factors in aging and DA neurodegeneration, little is known about the roles and regulation of Dot1l and H3K79 methylation in (DA) neurons. Here, we have investigated H3K79 methylation levels in the aged and diseased human midbrain and DA neurons and studied the gene-regulatory roles of Dot1l in the DA neurons of mice in depth.

We have found a consequent accumulation of H3K79 methylation in the neurons of the aged human brain. Moreover, we found a specific increase in lipofuscin-positive DA neurons, linking H3K79 methylation valence to pathology. Finally, we show in mouse models that in post-mitotic neurons the turnover of H3K79 methylation is very high, resulting in a major loss of the methylation marks (H3K79Me2/Me1) within 48 h. Finally, in the transcriptome profiling of DA neurons, we found Dot1l activity as an important repressor of respiratory chain genes next to positive effects on the level of DA-specific genes.

## 2. Results

**Figure 1:**
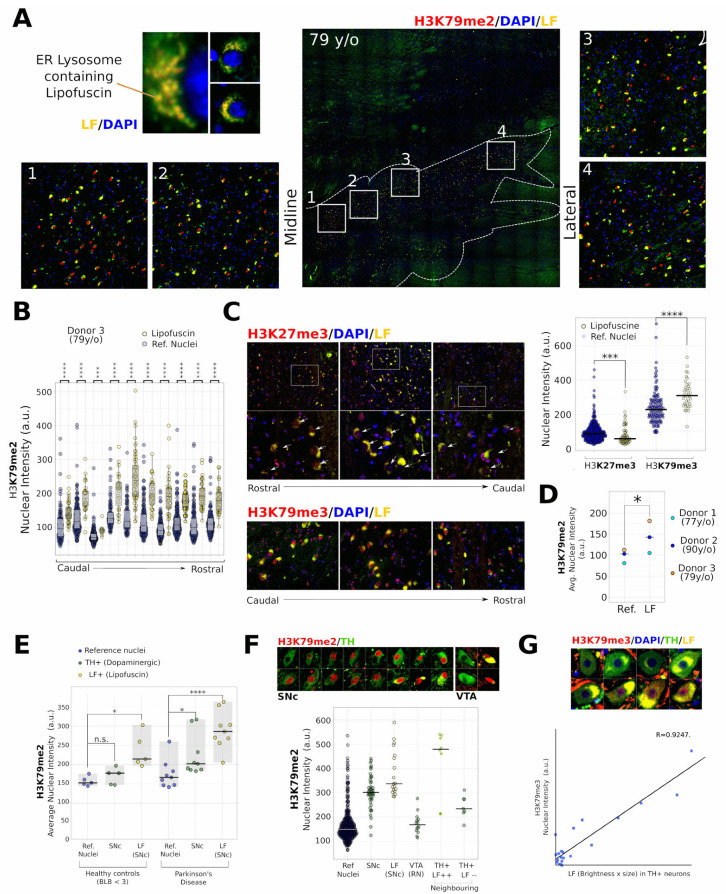
Increased H3K79me2 associates with Lipofuscin in the human midbrain. (**A**) Fluorescent image of typical red + green (yellow) autofluorescent lipid spheres containing accumulated dense protein (waste) structures in an ER-like morphology surrounding large nuclei (blue) (LF detail 40× lens, 10× lens overview, 1–4: 10× lens). Lipofuscin accumulation in a midbrain area overlapping with the SNc of a 79-year-old male with only minor loss of pigmented neurons, no Lewy body pathology (Supplementary Table S1), and no clinical PD but low TH levels (almost absent visible TH staining). White lines indicate the area with major LF accumulation. (A1–A4) Magnification boxes in numbered 1–4 representing regions from medial to lateral showing the correlation between LF-autofluorescent neurons and H3K79me2 nuclear staining. Nuclear staining of DAPI (blue), H3K79me2 (red), and in LF (autofluorescence green/red-yellow). (**B**) Quantification of sections from rostral to caudal. Each comparison shows LF-associated nuclei and automatically selected surrounding reference nuclei from a single merged image. Mann–Whitney statistical tests were performed to compare the median of LF-associated nuclear H3K79me2 staining with those of the surrounding reference nuclei for each photo. (**C**) Selection of images from a rostral to caudal series showing H3K79me3 (**bottom**), but not H3K27me3 (**top**), correlating to nuclei surrounded by LF. The upper right diagram shows the quantification of H3K27me3 and H3K79me3 of adjacent coupes in LF and surrounding reference nuclei. Mann–Whitney statistical tests were used for median (of average per nuclei) levels of H3K79me3 and H3K27me3 surrounding nuclei in adjacent coupes. (10× lens overview, 40× lens detail) (**D**) Comparison between average H3K79me2 levels of reference and LF-associated nuclei measured from combined rostral and caudal midbrain sections of three aged human donors. Paired Student’s t-tests was used to test if the average nuclear H3K79me2 levels increased as compared to surrounding reference nuclei in three different donors. (**E**) Comparison of dopaminergic (TH+) and surrounding LF+ in non-Parkinson’s and PD human donors in de substantia nigra pars compacta (SNc), compared to surrounding reference nuclei. Paired Student’s t-tests were used for statistics. (**F**) Further dissecting cell types of one individual with high levels of H3K79me2 staining in TH+ SNc neurons but not those of the ventral tegmental area (VTA), functioning as an extra internal control. The bottom diagram shows the individual nuclear average H3K79me2 levels of reference nuclei (Ref. Nuclei), TH+ neurons in the substantia nigra (SNc), surrounding LF+/TH low/absent (LF (SNc)), VTA neurons surrounding the red nucleus (VTA (RN)) and neighboring neurons that show at least minimal TH staining but then in the mere absence (TH+/LF−) or strong presence (TH+/LF++) of LF. (40× lens). (**G**) Correlation (Pearson) between the [size + intensity] of LF and nuclear H3K79me3 staining. (40× lens). Other statistics: * *p* = 0.05–0.01, ** *p* = 0.01–0.001, *** *p* = 0.001–0.0001, **** *p* < 0.0001. For (**E**), a post-hoc Bonferoni-correction for multiple testing was used.

H3K79me2 hypermethylation associates with lipofuscin in the aged human midbrain Lipofuscine (LF) and neuromelanin (NM) containing macro-vacuole structures have been proposed as markers for vulnerable DA neurons in the midbrain [28]⁠. In addition, the formation and maturation of Lewy bodies can involve stages of lysosomal dysfunction associating with lipofuscine accumulation [29]⁠. Densely folded autofluorescent protein aggregates in LF accumulate in the lipid lysosomal spheres, mostly the endoplasmic reticulum, emitting wavelengths of both 488 nm and 555 nm [30]⁠. As such, LF can be visualized as spherical yellow structures that surround nuclei (Figure 1A). Since LF does not overlap with nuclei (Figure 1A), we were able to investigate levels of histone modifications using immunological staining.

In a 79-year-old male donor without pathological and cognitive signs of PD, we found a large number of LF-surrounded nuclei stretching from the midline to the lateral which was associated with elevated H3K79me2 staining (Figure 1A). Nuclear quantification revealed a comparable pattern from the rostral to the caudal of elevated H3K79me2. (Figure 1B). We also investigated levels of the polycomb complex 2 histone mark H3K27me3. The reduction of H3K27me3 has been linked to senescent states and the up-regulation of neurodegeneration-associated genes [31,32]⁠. In contrary to H3K79me2, we have found that the average nuclear H3K27me3 staining was lower in the nuclei of LF-containing cells as compared to surrounding controls, while H3K79me3 was increased in sections adjacent to those used for H3K79me2 identification (Figure 1C and Supplementary Figure S1A,B), together suggesting that H3K79 hypermethylation, but not generally H3 hypermethylation, correlates to LF.

To investigate if the correlation between LF and H3K79 hypermethylation is a general phenomenon, we expanded our study with a 77-year-old and a 90-year-old donor, confirming a consequent increase in H3K79me2 in the rostral and caudal midbrain in all investigated sections (Figure 1D and Supplementary Figure S1C,D). This was regardless of the varying and scattered localization of LF+ cells. Overall, we only found remarkably few exceptions of LF+ cells, showing less excessive H3K79me2 (Supplementary Figure S1C,D). In summary, the LF-surrounded nuclei, including typical decondensed neuronal nuclei, have elevated levels of H3K79 methylation in aged individuals.

H3K79me2 hypermethylation in TH+ DA neurons of PD patients is not generally increased in the absence of LF.

Next, we investigated DA neurons and LF+ cells in the PD substantia nigra compacta (SNc). Again, we found, without exception, H3K79 hypermethylation correlating with LF, both in healthy and PD individuals (Figure 1E and Supplementary Figure S2). We also found an average increase in DA neurons from the PD patients. However, the effect was much less profound (Figure 1E). We did not find increased H3K79me2 in DA neurons in healthy individuals in the SNc, nor increased H3K79me3 in TH+ neurons without disease hallmarks (Figure 1E and Supplementary Figure S1E). An overview of the levels in individual nuclei per donor can be found in Supplementary Figure S2.

We further investigated one PD donor with high levels of H3K79me2 in TH+ neurons in more depth, by comparing the SNc DA neurons with those in the dorsal VTA which contained high levels of TH (Supplementary Figure S3). We did not observe H3K79-hypermethylation in TH+, even though the neighboring VTA LF+ cells and TH+ cells that contained LF showed similar patterns of a general increase relating to LF (Figure 1F). Finally, we want to point out that TH/LF double-positive cells were scarce, especially in the ventral tier of the SNc, even though we could find them in abundance in neighboring cells. In rhesus monkeys, the VTA contains more LF+TH+ neurons, while the accumulation of aging markers inversely correlates with TH [33]⁠. Indeed, we did find several scattered sites on the border of the SNc/VTA, more ventral to the red nucleus, where reasonable numbers of LF+ DA neurons were found in proximity. There, H3K79me3 (next to H3K79me2) correlated with the size and intensity of the LF-containing vacuoles (Figure 1G). In those TH+ cells, the levels of H3K79me3 only seemed to correlate to relatively high LF levels as compared to H3K79me2 in the previous datasets and generally in LF-containing cells/neurons (Figure 1G and Supplementary Figure S1, respectively.).

In summary, in all 17 human brain samples, and in each section that we have investigated, we have found H3K79me2 levels increased in cells that accumulated typical lysosomal LF in ER-like patterns. Only two individuals (both PD) had substantially elevated levels of H3K79me2 in the TH+ DA neurons in the absence of LF, while LF and TH-staining was scarce, but in those neurons, a similar correlation was found.

### 2.1. Neuronal Dot1l Transcript Abundance Increases through H_2_O_2_ and AMPK Activation

**Figure 2:**
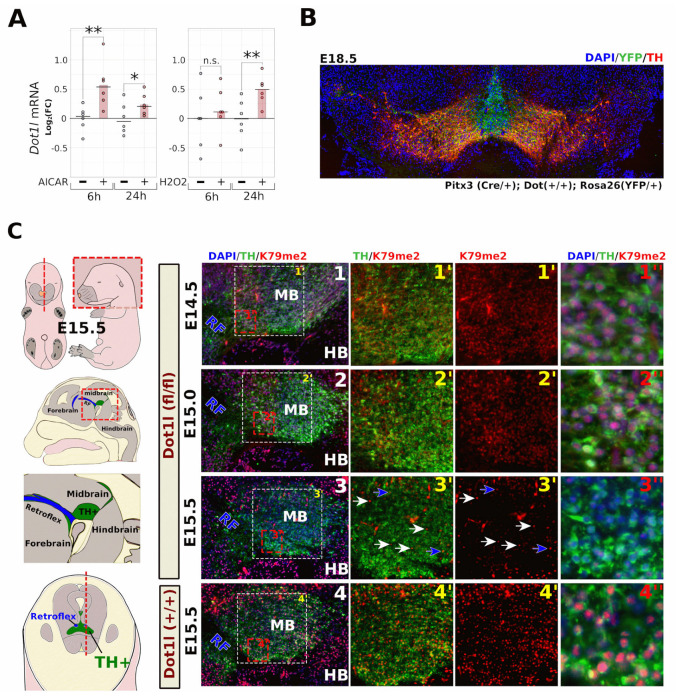
Neuronal *Dot1l* transcriptional regulation and H3K79me2 turnover. (**A**) Quantitative PCR of Dot1l mRNA levels in primary neurons at either 6 or 24 h following AICAR or H_2_O_2_ treatment with a final concentration of resp. 100 µm and 10 µm. starting at DIV11. Statistics: * *p* = 0.05–0.01, ** *p* = 0.01–0.001, n.s.: not significant. (**B**) Color-combined image of a coronal midbrain section of an E18.5 *Pitx3(Cre/+)*; *Dot1l(+/+)*; *Rosa26(YFP/+)* embryo, stained for TH (red), YFP (green), and DAPI (blue) (tiled image 10× lens). (**C**) In the left panel, a schematic representation of the location of the (RF), hindbrain (HB), and the DA (TH+) area at E15.5. In the right panel, a timeline of sagittal midbrain (MB) sections of *Pitx3(Cre/+)*; *Dot1l(fl/fl)* at E14.5 (C1), E15.0 (C2) and E15.5 (C3), and *Dot1l(+*/*+)* at E15.5 (C4) as control. All sections are matched by the presence of the retroflex (RF) and the location of the midbrain is marked MB. The first column (C1–C4) represents color-combined overviews (5× lens) of the whole midbrain with DAPI (blue), tyrosine hydroxylase (TH, green), and H3K79me2 (red). Columns 2 and 3 (C1′–C4′) are magnifications (10× lens) that represent TH/H3K79me2 (**left**) and solely H3K79me2 (**right**) staining. Column 4 (C1″–C4″) shows overlays at higher magnifications (40× lens) presenting the nuclear depletion of H3K79me2 at E15.5, specifically in the E15.5 *Pitx3(Cre*/*+)*; *Dot1l(fl*/*fl)* embryos (C3″). Blue arrows indicate the scarce TH+ neurons at E15.5 that still possess clear H3K79me2 staining, while white arrows indicate non-TH+ cells. These cells contain clearly visible H3K79me2 staining in the middle of low H3K79me2-stained TH+ neurons.

Several studies have suggested that cellular and mitochondrial stress may precede the up-regulation of Dot1l. As mentioned in the introduction, we wondered if these mechanisms are active in neurons too. To assess if bioenergetic manipulation or oxidative stress can influence neuronal Dot1l transcript abundance, we have treated primary neurons both with the AMP-mimicking AMPK activator, AICAR, and H_2_O_2_. After 6 h, Dot1l mRNA was significantly up-regulated in the primary neurons following the AICAR administration, while after 24 h, both H_2_O_2_ and AICAR showed increased Dot1l mRNA (Figure 2A).

### 2.2. Fast Turnover of H3K79 Methylation in Post-Mitotic DA Neurons

To further investigate Dot1l function and H3K79 dynamics in post-mitotic DA neurons, we crossed mice that had loxp-sites flanking exon 5 of *Dot1l* [34]⁠ with mice that started expressing Cre-recombinase from the Pitx3 locus in post-mitotic DA precursors [35],⁠ while activating a reporter allele (Rosa26-lox-stop-lox-YFP, Figure 2B).

Even though Cre is expected to be present around E12.5 [35]⁠, we did not observe reduced K79 methylation at E15.0 in *Dot1l* floxed neurons (Figure 2C). However, between E15.0 and E15.5, global H3K79 dimethylation rapidly declined in the majority of dopamine (DA) neurons (Figure 2C). Only most rostro-lateral (minority), and in the most medial sections, did we find cells that were still H3K79me2-positive (Supplementary Figure S4A,B). We expect those to be the youngest neurons that may still have sufficient Dot1l levels. Similar results were obtained for H3K79me1 at E15.5 (Supplementary Figure S4C,D), together suggesting a rapid global turnover of H3K79 methylation. At later embryonic stages (E18.5), both me1 and me2 remained absent in TH+ neurons, with only very sporadically a K79me1-positive DA neuron left (Supplementary Figure S1E).

### 2.3. Conditional Dot1l Deletion Leads to a Rostro-Lateral Non-Progressive Defect in Levels of Dopaminergic Markers

**Figure 3:**
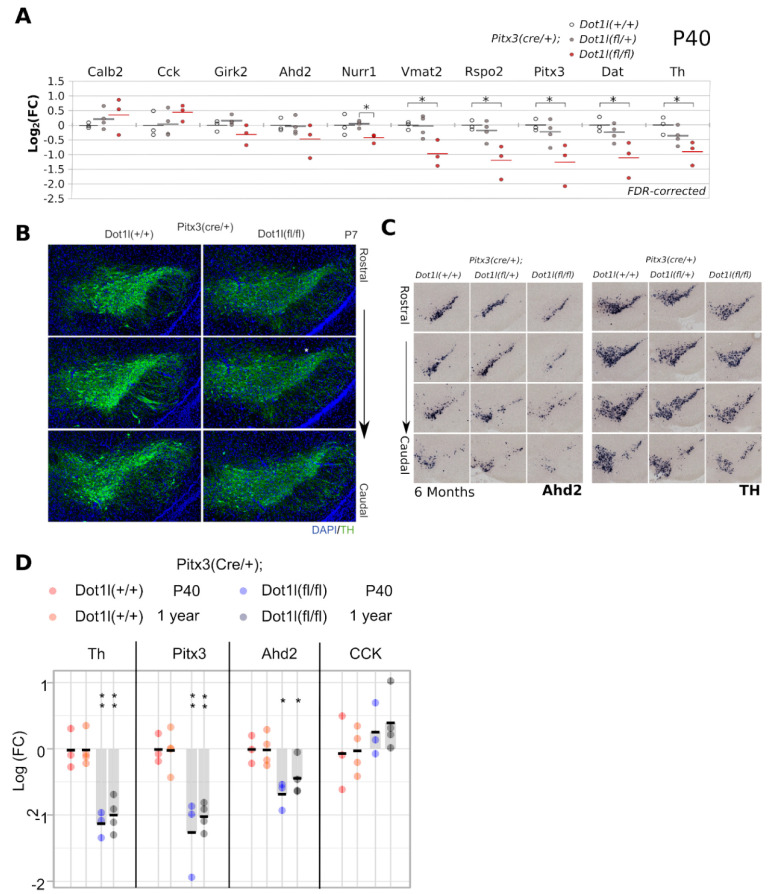
Neuronal defect in *Pitx3(Cre*/*+)*; *Dot1l(fl*/*fl)* mice. (**A**) Quantitative PCR of dopaminergic and midbrain (subset) markers on dissected P40 midbrains. White dots represent *Dot1l(+*/*+)* (n = 3), gray dots represent *Dot1l(fl*/*+)* (n = 4), red dots represent *Dot1l(fl*/*fl)* (n = 3), all in a *Pitx3(Cre*/*+)* background. Levels were normalized against *Tbp* transcript levels. (**B**) Immunohistochemistry for Th in P7 coronal sections of rostral *Pitx3(Cre*/*+)*; *Dot1l(+*/*+)* and *Dot1l(fl*/*fl)* mice. (10× lens) (**C**) In situ hybridization of *Ahd2* and *Th* transcripts in coronal slices of 6-month-old mice from more rostral to caudal, comparing *Dot1l(+*/*+)*, *Dot1l(fl*/*+)* and *Dot1l(fl*/*fl)*, showing especially in the latter clear reductions. (10× lens). (**D**) Quantitative PCR of dopaminergic and midbrain markers comparing p40 and one year old midbrains assessing mRNA levels of *Th*, *Pitx3*, the rostro/ventral marker *Ahd2*, and the dorso/caudal marker CCK similar as what observed in (**A**) and unchanged between p40 and 1-year-old Dot1l cKO mice. The red and orange dots represent *Pitx3(Cre*/*+)*; *Dot1l(+*/*+)* in P40 and 1-year-old mice, respectively. The blue and gray dots represent *Pitx3(Cre*/*+)*; *Dot1l(fl*/*fl)* mice. Additional statistics: a one-tailed Student’s *t*-test was used with * FDR-corrected *p* < 0.05.

In order to assess whether the removal of Dot1l would abolish the normal development of midbrain dopaminergic neurons, we analyzed several key DA markers in the region. With qPCR on dissected midbrains, we found that the transcript levels of critical DA genes were especially affected in full conditional Dot1l knock-outs (cKO) (*Th*, *Vmat2*, *Dat*, *Rspo2*, *Nurr1*, *Pitx3*, with *Girk2* and *Ahd2* following a trend), while heterozygous flox-mice were less affected (Figure 3A). In *cKO* mice at *P7*, we did not find large structural abnormalities, although the level of Th seems to be lower in all sections analyzed compared to WT (Figure 3B), supporting the initial qPCR data. *Th* and *Ahd2* in situ hybridization (ISH) analyses of 6-month-old mice showed that small structural changes or expression loss are present in the substantia nigra. (Figure 3C). Finally, in order to analyze whether there is a progressive loss of DA markers we compared mRNA levels of critical DA genes between p40 and one-year-old *Dot1l(fl/fl)* mice (Figure 3D). The data suggest an initial loss of expression of these DA genes; however, no further progressive change could be detected.

### 2.4. Dot1l Ablation Leads to Increased Mitochondrial Respiratory Chain Transcripts

**Figure 4:**
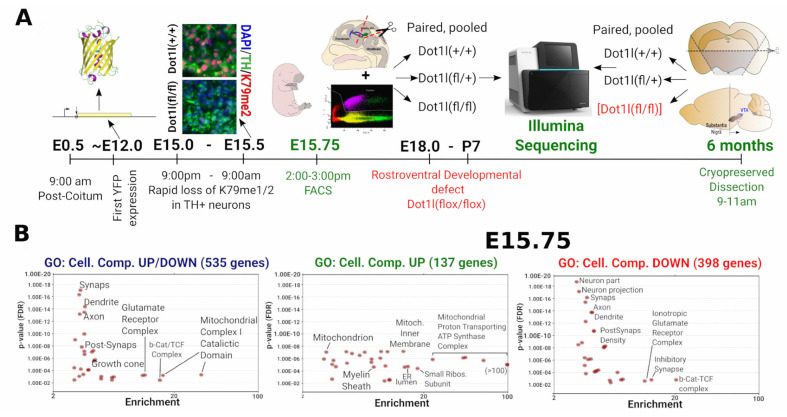

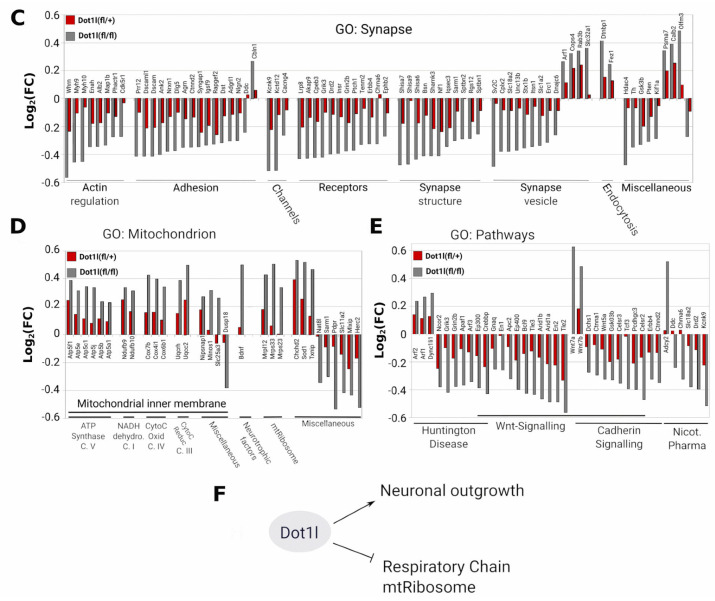
Transcriptome analysis of E15.75 DA neurons with reduced Dot1l/H3K79me2 levels. (**A**) A timeline overview of the set-up. At E12.0, the first YFP expression appears. Between E15.0 (9:00 p.m.) and E15.5 (9:00 a.m.), most of H3K79me1/2 is strongly reduced. At E15.75 (~3:00 p.m.), the neurons were sorted (FACS) based on Cre-induced YFP expression. Per each pregnant female, only three embryos (each genotype condition) were used to ensure correct pairing and reduce variation. Per sample, three or four embryos were pooled to generate three conditions of n = 3 that were used for Illumina sequencing. (**B**) Cellular component analysis (Panther) of the total 535 genes (398 down/137 up) were found deregulated (*p*-Adj. < 0.05) following a DEseq2 analysis for differential expression, Dot1l(+/+) versus Dot1l(fl/fl). Total or solely up- and down-regulated genes, as a threshold for Panther gene-ontology, were set a minimal enrichment of threefold and a *p*-value < 0.01. (**C**) The 2Log (fold change) of differential expressed genes (Dot1l(+/+) versus Dot1l(fl/fl)) of the most prominent GO terms found in: Synapse (**C**), Mitochondrion (**D**), and an overview of GO pathways (**E**). In (**F**), an overview of the key post-mitotic DA gene-regulatory roles for Dot1l. In DA neurons, at physiological levels, Dot1l activates neuronal outgrowth while repressing ribosomal and mitochondrial transcripts.

The conditional deletion of Dot1l (Pitx3-Cre*Dot1l-Floxed) and transcriptome approach is schematically presented (Figure 4A) Firstly, we investigated young (E15.75) *Pitx3(Cre*/*+)*; *Dot1l(fl*/*+)* and *Dot1l(fl*/*fl)*; *ROSA-lox-STOP-lox-YFP*, shortly after the loss of the H3K79Me2 mark (Figure 2C) by FACS GFP-positive cells. Since the median half-life of transcripts is estimated at 7–9 h, and those of proteins ~48 h [36,37]⁠, we reasoned that FACS sorting neurons ~5/6 h after global loss of H3K79me2 would provide us with the initial transcriptional changes as a result of that H3K79me2 loss. Deseq2 analysis of E15.75 ablated Dot1l DA neurons showed 535 genes significantly deregulated (Figure 4B). We next performed a Panther cellular component analysis [38]⁠ and found genes involved in neuronal outgrowth, Wnt-related (Beta Catenin binding), and mitochondrial (mt) genes being deregulated (Deseq2 Adj. *p*-value < 0.05 in *Dot1l(flox*/*flox)*). Surprisingly, separating the total deregulated genes into up- *and* down-regulated, especially genes related to mitochondrial function were up-regulated, while the genes involved in neuronal outgrowth and Wnt-regulation were down-regulated, with the exception of the Wnts: Wnt7a and Wnt7b (Figure 4B–E). Further analysis of the GO term ‘Synapse’ revealed that the only exceptions were genes involved in vesicle regulation and endocytosis, the large majority of genes related to synapse function were down-regulated (Figure 4C). Analysis of the GO term ‘Mitochondrion’ showed, especially in genes of the respiratory chain, the mitochondrial inner membrane in general, and the mtRibosome being up-regulated (Figure 4D). Interestingly, we found similar changes in the heterozygote Dot1l mice. This suggests that the aberrations found are the direct consequence of the level of Dot1l activity, and as such, represented by the level of H3K79 methylation. Taken together, in maturing DA neurons, genes involved in neuronal function outgrowth are generally activated by Dot1l and/or H3K79 methylation, next to Wnt signaling (Figure 4E). Importantly, many genes belonging to the family of the respiratory chain are repressed (Figure 4F). This suggests that Dot1l activity (H3K79 methylation) is repressive for the electron transport function and beneficial towards other neuronal functions. (Figure 4F).

### 2.5. Respiratory Chain Transcript Abundance Remains up in 6 Months Old Neurons with Reduced Dot1l

**Figure 5:**
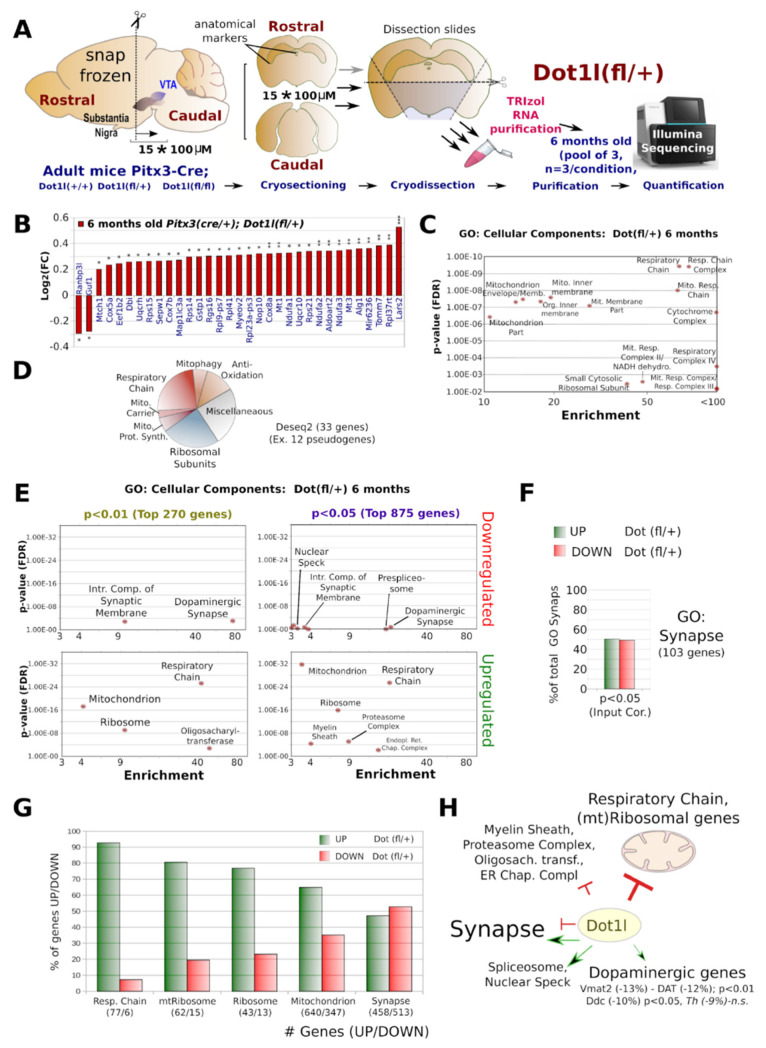
Transcriptome profiling of 6-month-old *Pitx3(Cre*/*+)*; *Dot1l(fl*/*+)* mice. (**A**) Schematic overview of the dissection and transcriptomics approach. Snap frozen 6-month-old mice brains were sliced in a cryostat until rostral anatomical markers of the midbrain were reached. Exactly 15 slices, 100 µm thick, were sliced and the ventral midbrain area was further dissected by removing the dorsal midbrain and hemicortices, which were collected and thawed immediately into Trizol. Three (n) of paired-pooled samples were generated for each of the three conditions (*Pitx3(Cre*/*+)*; *Dot1l(+*/*+)*, *(+*/*fl)* or *(fl*/*fl)* and the pooled samples were Illumina sequenced and analyzed following DEseq2 statistics. (**B**) Bar diagram of all DEseq2 *p*-Adj. *p* < 0.05 significantly deregulated genes presented as Log2 fold change. (**C**) Panther cellular component analysis of the Deseq2 differential expressed genes (cut-off: minimal enrichment 3, *p* < 0.01). (**D**) Gene function diagram of all DEseq2 deregulated genes. (**E**) Cellular component analysis of top down- (**upper diagrams**) and up-(**bottom diagrams**) regulated genes with either a non-adjusted *p* < 0.01 (**left diagrams**) or *p* < 0.05 (**right diagrams**). Performed with Panther cellular component analysis (minimal enrichment 3, *p* < 0.01). (**F**) Bar diagram representing the percentage of GO: Synapse genes (103 in total) being up- or down-regulated when retrieved from *p* < 0.05 (top 875 genes) after correction for the slightly over-represented number of up-regulated genes within this group (376 down/499 up). (**G**) Bar diagram of bottom-up analyses of up versus down-regulation of total RC, mtRibosome, Ribosome, Mitochondrion, and Synapse related genes. (**H**) Graphical summary of transcriptomic changes and Dot1l targets in DA neurons with especially respiratory chain genes are up-regulated in heterozygous *Dot1l* floxed DA neurons, suggesting a repressive function of Dot1l. Statistics: *** represent DEseq2 Adj. *p* < 0.001, ** *p*< 0.01, and * *p* < 0.05.

In order to further confirm the discovered up-regulation of electron transport chain genes in developing DA neurons (Figure 4), we decided to perform transcriptome analysis on 6-month-old mice with special emphasis on heterozygous Dot1l animals (Figure 5). The approach and isolation methods are represented in Figure 5A. DEseq2 analyses showed 33 genes and 12 pseudogenes differential expressed (*p*-Adj. < 0.05), of which only two were down-regulated (Figure 5B,D). Panther analysis of these genes showed that, especially for genes encoding components of the respiratory chain, the ribosomal subunits and mitochondrial regulators were increased (Figure 5C). Almost half of the validated genes were mitochondrial, including the down-regulated gene *Guf1* (Figure 5B), which is a known regulator of the respiratory chain, though, especially in conditions of cellular stress [39]⁠. Next to mitochondrial genes, among which those involved in anti-oxidation, mitophagy, protein syntheses, and a carrier, a quarter of the dysregulated genes encoded ribosomal subunits (Figure 5D), suggesting a broad role of DOT1L in mitochondrial and ribosomal gene regulation in these adult neurons (Figure 5C,D).

Since we used dissected midbrains, the effect size of deregulated genes ubiquitously transcribed is limited because floxed neurons only comprise a portion of the dissected region. Therefore, we have analyzed also larger groups of 270 (*p* < 0.01, non-adjusted) and 875 (*p* < 0.05, non-adjusted) genes (Figure 5E). These larger groups are more equally divided between up- and down-regulated genes (Supplementary Figure S5A), presumably as a result from especially the up-regulated (mitochondrial genes) being the primary group with the largest effect size, revealed in the Deseq2 analysis. However, deregulated synaptic genes were not as consistently down-regulated as at E15.5 (Figure 5F).

We found very similar outcomes with GO, regardless of the statistical cut-off; mostly up-regulated respiratory chain and ribosomal genes (Figure 5C–E). Interestingly, with this GO approach, we did find groups of DE genes involved in myelin sheath formation, the proteasome, and ER chaperones among the up-regulated genes (Figure 5E).

With our approach, the effect size following down-regulation may be slightly underestimated for ubiquitously expressed genes. However, this is not the case for specific dopaminergic genes. Especially DA transporters, cholinergic receptors, and genes involved in DA synthesis will have comparable effect sizes between up- anddown-regulation, though, were only relatively slightly down-regulated, with the rate-limiting DA-producing enzyme, tyrosine hydroxylase (Th), not among the 875 significantly deregulated (i.e., the top 376, *p* non-adj < 0.05, down-regulated) genes. In addition, this is in line with our qPCR analysis at P40 and the ISH analyses at P7 and 6 months (Figure 3) and the minor effects observed in the heterozygous *Dot1l(fl*/*+)* animals at E15.75 (Figure 3D). Opposite to the developmental E15.75 stage, the top deregulated synaptic genes at 6 months heterozygous *Dot1l* DA neurons were slightly more often up-regulated, even after correction for the over-represented number of up-regulated versus down-regulated genes in this group (Figure 5F and Supplementary Figure S5A).

Finally, we also analyzed the dataset using a bottom-up approach by selecting known genes that encode components of the respiratory complex (RC) and the (mt)ribosome, synapse (GO:0045202), or mitochondrial (MitoCarta 2.0) genes in general. We found most of the RC (93%), mtRibosome (81%), and ribosome (77%) genes up-regulated on average, with even 65% of the total 987 mitochondrion related transcripts retrieved from our dataset (cutoff minimal 80 read-counts) being up-regulated (Figure 5G). Of the retrieved synaptic genes (971) was slightly more deregulated towards the downside (53%, Figure 5G). In Figure 5H, the transcriptional roles of Dot1l in the DA neurons are schematically summarized.

### 2.6. mtDNA Encoded Genes and a Selection of Genes Involved in Mitochondrial Morphology Form Exceptions

**Figure 6:**
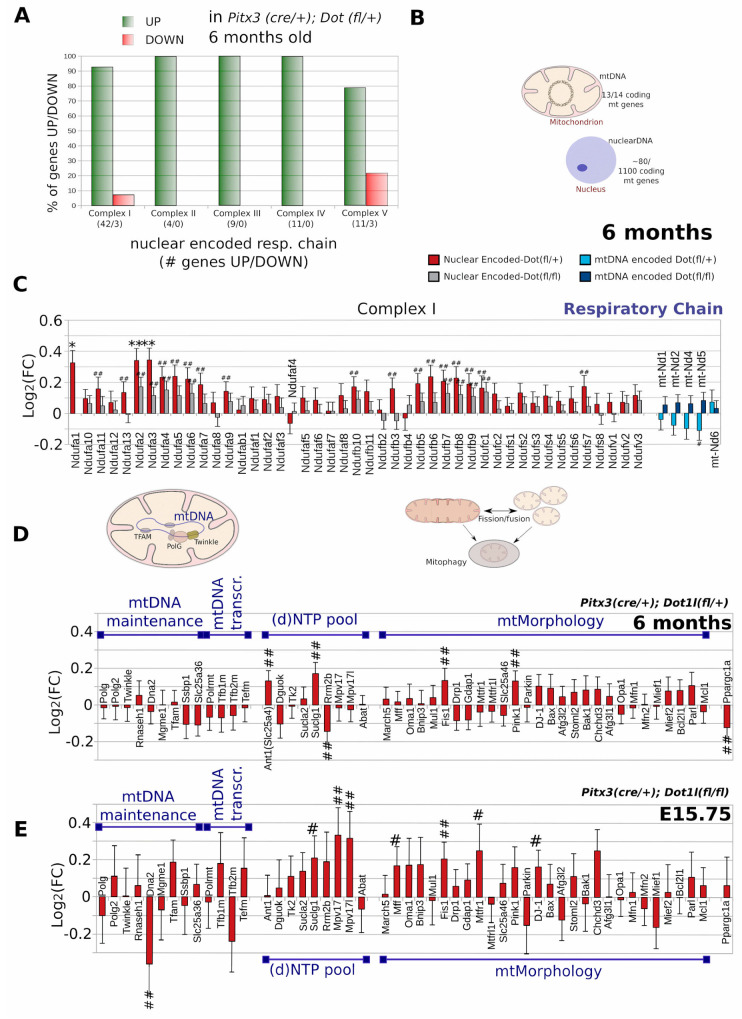
Majority of respiratory chain genes, *Pink1*, *Fission*, and *DJ-1* show upward trend following Dot1l reduction. (**A**) Ratio of genes up- versus down-regulated in heterozygous *Dot1l* floxed DA neurons. (**B**) Schematic overview of mitochondrial and nuclear DNA, indicating an approximation of the number of (encoding) mitochondrial genes located at the mtDNA and nuclear DNA. Respiratory chain genes are encoded both at the mtDNA (13 out 14 encoding genes) as well as the nucleus (~80/1100 mitochondrial genes). (**C**) All retrieved complex I respiratory chain genes of 6-month-old mice. For nuclear-encoded genes, red bars represent (*Dot1l(fl*/*+)*, gray bars represent *Dot1l(fl*/*fl)*, and for mtDNA encoded genes: light blue bars represent (*Dot1l(fl*/*+)*, dark blue bars represent *Dot1l(fl*/*fl)*. (**D**,**E**) Selection of genes involved in mtDNA maintenance, mtDNA transcription, maintenance of the (d)NTP pool or mtMorphology, either *Dot1l(fl*/*+)* for 6 months (**D**) or *Dot1l(fl*//*fl)* for E15.5 (**E**), red bars represent *Dot1l(fl*/*+)*, gray bars represent *Dot1l(fl*/*fl)*. Statistics: ** represent DEseq2, *p* < 0.01 and * *p* < 0.05; # represent non-Adj. *p* < 0.01 (Wald-test), ## *p* < 0.05 (Wald-test).

While RC components, including those coded on the mtDNA (Figure 6A), provided that they had passed our experimental pipeline (i.e., ND1,2,4,5;mtCyb;Cox1—*HeavyStrand*, ND6—*LightStrand*), were near completely up-regulated at E15.75 (Supplementary Figure S5B), as near all mtRibosomal subunits were found to be up-regulated (Supplementary Figure S5C). However, in 6-month-old mice there are a few exceptions of complex I and complex V members (Figure 6A and Supplementary Figure S6A). In particular, while nuclear-encoded complex I members of the RC and ribosome are still generally up-regulated, those encoded at the mtDNA are down-regulated in the heterozygous mutant (Figure 6B and Supplementary Figure S6B).

We also included the cKO transcript profile here showing that, despite the reduction of neurons, RC genes are generally more abundant than the controls (Figure 6B).

Prompted by the broad deregulation of mitochondrial genes, we selected a set of known critical regulators related to PD and mitochondrial health, such as genes involved in mtDNA maintenance and mtMorphology, to seek if a shift in mitochondrial maintenance pathways could be observed (Figure 6C,D). Interestingly, in 6-month-old heterozygous Dot1l cKOs, *Fission1* and *Pink1*, but not Drp1 for instance, all key factors that regulate mitochondrial morphology, mitophagy, and the import of transcripts into mitochondria [40]⁠ were up-regulated (*p* ≤ 0.01, non-adjusted) (Figure 6C). Instead, *Pgc1-α* (*Ppargc1a*), a key regulator of mitochondrial respiratory genes and activator of mitochondrial biogenesis was among the top 270 deregulated genes (*p* ≤ 0.01, non-adjusted) (Figure 6C,D) [41]⁠. Surprisingly, *Pgc1-α* is not down-regulated at E15.75 in full Dot1l cKOs (Figure 6E).

## 3. Discussion

Dynamic epigenetics and transcript profiles may be pivotal in age-related diseases that involve the loss of neurons and their functions. Here, we have studied DOT1l and H3K79 methylation in human and mouse midbrains, and specifically, dopaminergic neurons, in an attempt to study their potential as epigenetic and gene-regulatory key players in neuronal aging and disease processes. DOT1l has been indirectly coupled to aging-related processes by its association with epigenetic mechanisms that involve Sirt proteins, known regulators of bioenergetics [15,16,17,18]⁠. More recently, data suggests that Dot1l itself may have prolonged deregulation in neurons following early life stress [23]⁠. Our findings further support the idea that (1) H3K79 methylation can be highly dynamic in neurons, especially in maturing neurons, (2) that transcription of Dot1l is regulated by energetic and oxidative challenges in neurons, (3) that Dot1l is a master regulator of mitochondrial and ribosomal genes in dopaminergic neurons at physiological levels, and, finally, (4) that in the aged human midbrain and substantia nigra (SNc), the accumulation of lipofuscin (LF) consistently correlates with H3K79 hypermethylation.

### 3.1. The Relation between Dot1l and H3K79me2 Deregulation and Cellular Stress

Although evidence for a causal role in the deterioration of neuronal function or PD is scarce, LF has been related to lysosomal dysfunction, ER stress, mitochondrial turnover, and oxidation, and is generally accepted as an aging hallmark that may play a role in the early stages of Lewy body formation [29,42]⁠. Our findings combined, we hypothesize that increased levels of oxidation may increase H3K79 methylation in neurons. In neurons accumulating LF, a constant state of oxidation may be registered and signaled by cell-intrinsic pathways, causing a shift towards a (more) catalytic state of Dot1l that also links with mitochondrial dynamics. We can see Dot1l as an intermediator of this process. It seems likely that the observed H3K79 hypermethylation represents a chronically shifted epigenetic equilibrium, potentially being more harmful instead of guarding cellular homeostasis.

Neurons that do not succeed in breaking down aggregates and (macro)vacuolar structures may chronically register a state of oxidation. We can imagine that high levels of H3K79me2, which are associated with LF, are the end state of such a process of, among other effectors, chronically increased Dot1l activity.

Reduced counteracting mechanisms, for example, a reduction of histone turnover following reduced H3.3 expression, could further add to the shift in methylation states, with H3K79me2 residing on the portion of H3.3 subunits that may turnover most rapidly in younger neurons, a process that has been suggested to influence the H3K79 methylation state as well [2,43]⁠. Moreover, histone turnover can be a more prominent effector at histones marked by active marks, which could explain why H3K27me3 does not accumulate in LF-associated nuclei. However, if we compare the rapid loss of post-mitotic neuronal H3K79me2 following floxed *Dot1l* alleles, we would not be surprised if demethylation enzymes, such as the recently described H3K79 demethylase KDM2B [44]⁠, further add significantly to the equation. At least, the fast global turn-over of H3K79 methylation in post-mitotic neurons suggest different dynamics than H3K27me3, which takes approximately 3–4 weeks after cKO of methyltransferase activity [31]⁠.

Another point worth discussing is the divergent and, on average, only mild increase of H2K79me2 in Parkinson’s disease (PD), often lower than the surrounding LF-containing neurons or compared to TH neurons that contain LF. Whether the two individuals with higher levels in TH+ cells are exceptions with an atypical form of PD, simply suggesting that H3K79 hypermethylation is a rare phenomenon in DA neurons, or if most SNc DA neurons are too vulnerable to survive (or express TH) with a substantial LF/H3K79me2/3 burden, remains uncertain. Interestingly, in aging rhesus monkeys, LF associates especially with VTA DA neurons and less with those of the SNc [33]⁠. This is could be the consequence of subtypes of DA neurons, mostly found in the VTA, still expressing TH while accumulating LF. Since we have found many LF encircled large nuclei in the middle of the substantia nigra, surrounded by TH+ neurons with similar large nuclei and macro morphology with clusters and similar relative distance (observational), we can imagine that (some) of the strongly LF+ neurons are used to express TH. It would be interesting to study the relationship between TH levels and LF or lysosomal defects in more depth since ultimate attempt to clear neurons from dysfunctional organelles and aggregates may undesirably affect functional proteins, such as TH.

Another explanation would be that SNc DA neurons develop LBs, instead of LF, in times of stress, perhaps as a consequence of the large number of mitochondria, of which (near-)complete units form the main components of matured LBs [29]⁠.

### 3.2. Influencing Dot1l Activity to Alleviate Aging Mechanisms and Neuronal Pathology

The broad down-regulation of nuclear-encoded respiratory genes is a transcriptional hallmark of PD [41]⁠. However, attempts to improve (mitochondrial) homeostasis in PD-models by over-expression of PGC1-α (Ppargc1a), a key inducer of mitochondrial biomass, have not been successful and may even worsen the disease [45]⁠. However, in several ways, the manipulation of overall mitochondrial transcription through Dot1l will differ from overexpression of PCG1-α. Firstly, whereas, hypothetically, PGC1-α needs to be overexpressed, Dot1l activity would need to be down-regulated to ensure a rescue of respiratory genes. Secondly, lower levels of Dot1l have been shown to slightly lower PGC1-α levels (Figure 6), possibly resulting in increasing the respiratory chain and anti-oxidation transcription and inner membrane-complex-turnover in the absence of increased mitochondrial mass or fusion. Moreover, genes related to the proteasome were increased (Figure 5H), which may alleviate the burdens of cellular waste. In summary, lowering DOT1L activity has the potential to alleviate some of the cellular burden that is associated with aging and neuronal pathologies. Future experiments with specific cellular stressors should shed light on the causal relationship between higher levels of H3K79 methylation levels and cellular resilience levels of dopaminergic neurons.

Finally, another interesting broad regulator of RC genes is the mitochondrial translation elongation factor GUF1, the only mitochondrial factor that is significantly reduced at 6 months (Figure 4B). Ablation of the highly conserved *C. elegans* homolog of GUF1, mtEF4, protects against paraquat toxicity in *C. elegans* [46]⁠. Next to this, the conserved yeast GUF1 regulates the RC and ribosomes under stressed conditions [39]⁠.

### 3.3. Transcriptional Exceptions: mtDNA Encoded Respiratory Genes

Although few transcriptional or epigenetic mechanisms seem able to compensate completely for the transcriptional consequences of Dot1l reduction, the two paralogs, Atp5g1 and Atp5g3, form exceptions involved in complex 5 abundance (Supplementary Figure S6A,B). This is also the case for mtDNA-encoded genes that seem to be decreased in heterozygous cKOs, while even slightly increased on average in *Pitx3-Cre*; *Dot1l(fl*/*fl)* full cKOs (Supplementary Figure S6B). Remarkably, a similar discrepancy between nDNA and mtDNA transcriptional deregulation has been observed in a recently published study performed by NASA on the health of astronauts during spaceflights [47]⁠. Cells that underwent a period of time in space showed nDNA respiratory transcripts that were broadly reduced, similar to PD [41]⁠, while mtDNA encoded transcripts were up-regulated [47]⁠.

### 3.4. Regulation of Mitochondrial Turnover and Mitogenesis

If no transcriptional compensation of mitochondrial regulation abnormalities can be found then increasing the turnover of mitochondrial components, or whole mitochondria may be another way for cells to avoid a mitochondrial overload following Dot1l dependent ‘mtComponent’ deregulation. Indeed, we find regulators of mitochondrial morphology and turnover, such as *Fis1*, *Pink1*, and *Dj1* [40], up-regulated both at E15.75 and in 6-month-old mouse Dot1l cKO models (non Adj. *p* < 0.5/*p* < 0.1) (Figure 6D,E), as well as proteosomal components. This is contrary to the stress-regulated mitophagy pathways that include *Drp1⁠*, which is slightly up-regulated on average at E15.75, but down-regulated at 6 months.

In summary, Dot1l activity, and therefore H3K79 hypermethylation, may be regulated by and associated with states of neuronal stress in human neurons. The fast turnover of H3K79 methylation and apparent dynamic regulation of Dot1l activity in neurons suggests an adaptable mechanism with Dot1l as a central master repressor of mitochondrial transcripts.

## 4. Methods and Materials

### 4.1. Human Tissue and Anatomical Selection

Human donor specifications are provided in Supplementary Table S1 (‘Experiment 1’: Figure 1A–D,G) and Table S2 (‘Experiment 2’: Figure 1E,F) and were obtained from the Netherlands Brain Bank (NBB) as paraffin blocks of hemi-midbrains (Supplementary Figure S3A). The rostral to caudal tissue sections were collected from two or three adjacent paraffin tissue blocks for each donor (as provided by the brain bank). Slices were matched by determining the position where the first most rostral, low vulnerable TH+ VTA neurons flanked the dorsal side of the red nucleus using TH-DAB stainings (Supplementary Figure S3B,C). Experiment sections proximate but caudal to this position have been used; retrieved from one mid-DA system paraffin block for each donor.

### 4.2. Immunohistochemistry Human Brain Tissue

Deparaffination/rehydration was performed in: 3 × 4′ in xylene, 2 × 4′ 100% ethanol, 2 × 4′ in 96% ethanol, 4′ in 80% ethanol, 4′ in 70% ethanol, and 4′ in 50% ethanol. Then slides were washed twice for 5 min in PBS and incubated for 5 min in 0.01 M citrate buffer at RT. Antigen retrieval was performed in 0.01 M citrate buffer by heating samples for 2 min at 800 W approaching boiling point. Subsequently, an 85–93 °C temperature was maintained by giving short 200 W bursts for 20′ with increasing intervals. Slides were subsequently incubated in a 67 °C stove for 60′. After cooling down the slides in a water bath, they were washed in TBS for 5′ and then blocked for 60′ with 5% NDS in THZT. Slides were washed 2 × 5′ in TBS and then incubated in sheep-α-TH (1:500, Millipore, Burlington, MA, USA, Ab1542) overnight. Then, slides were washed 3 × 5′ in TBS and incubated in donkey-α-sheep-488 (1:500, Life Technologies, Carlsbad, CA, USA, A11015) in TBS for 90′. Slides were subsequently washed 3 × 5′ in PBS and blocked in 5% FCS in THZT for 60′. Slides were then washed again in 2 × 5′ PBS and incubated overnight in rabbit-α-H3K79me2/3 or H3K27me3 in THZT. Subsequently, slides were washed in 3 × 5′ PBS and incubated in goat-α-rabbit-555 (1:1000, Life Technologies, A21207) in PBS for 90′. Slides were then washed in 2 × 10′ in PBS and subsequently incubated in DAPI (1:3000) in PBS for, 5′ in experiments 1, 8′ in experiment 2 (optimized for large neuronal nuclei). Finally, one PBS was washed for 5′ and embedded in Fluorsave (Millipore, 2848323).

### 4.3. Imaging

Microscope imaging for analysis of IMHC experiments was performed using a Leica DFC310 FX microscope with Leica MM AF 1.6.0 software. For human tissue, 2 × 2 or 4 × 4 images were generated at a 40× magnification of the SNc for each slice. For experiment 1, to assess the rostral to caudal LF associated nuclear staining, 10 images were generated from three separate tissue blocks, originating from a 79-year-old donor. For the additional donors, two photos originated from two slices from the 90-year-old and 77-year-old donors. In experiment 2, the images generally contained 5–50 TH+ neurons per image from lateral to medial. In total, 2 × 3 images were generated from two slices separated by 160 µm and spread from lateral to medial. VTA DA neurons were captured in small groups or as single neurons surrounding the red nucleus since they are more scattered over a larger area. The location and subdivision of the VTA area was based on Root and colleagues [48]⁠.

### 4.4. Quantification of IMHC Signal Humane Tissue

Quantification of H3K79me2/3 staining in TH+ DA neurons was performed using ImageJ (v149) and blinded (imaging and quantification were independently and blinded performed by two individuals). Reference nuclei and the nuclear area was determined by converting the DAPI signal to binary images, and selecting nuclei regions of interest (ROIs) at size (2000–6000 pixels) and circularity > 0.7, selecting and labeling the majority nuclei in experiment 2. Next, the regions of interest were overlaid with color combined to separate DA (TH+) with/without the inclusions or LF-associated neurons. For experiment 1, LF+ nuclei ROIs were manually selected and, opposite, manually deselected from the reference nuclei set. ROIs were overlaid with 16-bit H3K79me2 image layers to quantify the average signal in each nucleus. In experiment 2, this was followed by a color-combined image overlay to select DA neuronal nuclei neurons, those with/without LF or black pigmented inclusions. H3K79me2 levels of LF+ were manually validated in experiment 2. We found levels of H3K79me2 low nuclei were lower than background of some fiber structures that were not equally divided between and within pictures, which prompted our decision to perform calculations as represented in Figure 1, without the background correction. In Supplementary Figure S2, the individual measurements with/without the local background correction have been presented as well (which showed no statistical difference). For this, we have selected >10 areas from each photo, in between cells in low nuclear regions but avoiding fiber tracts depleted from cells but with high local levels of background to determine the average background for each photo which were used for individual background correction (IBC) by subtraction. As a comparison, an equal background correction (EBC).

### 4.5. Animals

The C57BL/6J mouse line was used as background strain (Charles River). Mice expressing Cre from the *Pitx3* locus and Cre-induced YFP from the *Rosa26* were previously described [35]⁠ and crossed with floxed *Dot1l* mice that were a kind gift from Dr. Zhang [34]. *Pitx3(+*/*+)*; *Dot1l(fl*/*+)*; *Rosa26(YFP*/*YFP)* breeding and *Pitx3(Cre*/*Cre)*; *Dot1l(fl*/*+)*; *Rosa26(+*/*+)* animals were crossed to generate offspring containing all experimental genotypes. All procedures were according to and fully approved by the Dutch Ethical Committees for Animal Experimentation (University of Amsterdam, Amsterdam, The Netherlands).

### 4.6. Immunohistochemistry Mouse Brain Tissue

PFA sucrose-preserved brains were fixed as whole heads for 4 h (E16 or younger), or as whole brains for 7 h, in 4% paraformaldehyde (PFA) at 4 °C, put in PBS containing 30% sucrose (*w*/*v*) until they sunk, and then kept at −80 °C until sliced at 16 µm sections using a cryostat. To perform IHC, the slides were thawed, washed with PBS (3×), post-fixated with 4% PFA for 10 min, and then washed with PBS (3×) again. Using antibodies against histone marks, antigen retrieval was performed by incubation for 5 min in 0.01 M citrate buffer at RT, heating in 0.01M citrate buffer by heating samples for 2 min at 800 W in the microwave to ±90 degrees, followed by 200 W pulses for 20′ and incubated at 67 degrees for 1 h. After cooling the slides down, they were blocked with 5% NDS in THZT, washed in TBS (3×), and incubated in sheep-α-Th overnight. The next day, they were washed with TBS (3×) before incubation with donkey-α-sheep-488 in TBS for 90 min, washed in PBS (1×), blocked in 5% FCS in THZT for 1 h, washed in PBS (2×) and incubated o/n with rabbit-α-H3K79me1/2 in THZT. The following day, slides were washed in 3 × 5′ PBS and incubated with Goat-α-rabbit-555 in PBS for 90′. Slides were then washed in 2 × 10′ in PBS and subsequently incubated in DAPI in PBS for 7′. Finally, slides were washed once again in PBS for 5′ and embedded with Fluorsave (Millipore, 2848323). Microscope imaging for analysis of IMHC experiments was performed using a Leica DFC310 FX microscope with Leica MM AF 1.6.0 software.

### 4.7. Primary Neuronal Culturing and Treatment

Twelve wells plates were coated o/n with poly-L-lysine (0.1 mg/mL [SigmaAldrich, St. Louis, MO, USA]) in 0.1 M borate buffer pH 8.5 (0.04 M Boric acid [Merck Millipore], 0.01 M Borax [SigmaAldrich]). A total of 1 mL of plating medium (MEM Eagle’s with Earle’s BSS medium [Invitrogen, Waltham, MA, USA], supplemented with 10% heat-inactivated FBS [Gibco, Waltham, MA, USA], 0.45% (wt/vol) glucose [Merck Millipore], 100 mM sodium pyruvate [Invitrogen], 200 mM glutamine [Invitrogen] and penicillin/streptomycin [Invitrogen]) was added to each well and the plates were stored in the incubator on 37 °C with 5% CO_2_. Primary cortical cultures were prepared from E17.5 C57BL/six mice. E17.5 cortical tissue was dissected in an ice-cold dissection medium (HBSS medium [Invitrogen], supplemented with 100 mM sodium pyruvate [Invitrogen], 20% (wt/vol) glucose [Merck Millipore] and 1 M Hepes [SigmaAldrich]) washed and trypsinized at 37 °C for 20 min and 100 µL of DNase [Thermo Fisher Scientific, Waltham, MA, USA] was added for 5 min before dissociation by trituration. 1 ML plating medium containing 100,000 cells/mL were added to each well of a 12-wells plate containing 1 mL of pre-incubated medium. After 2 days, 50% of the medium was replaced with a maintenance medium (Neurobasal medium [Invitrogen] supplemented with 2% B27 [Invitrogen], 200 mM glutamine [Invitrogen] and penicillin/streptomycin [Invitrogen]), and 10 µm 5-Fluoro-2′-Deoxyuridine (Fudr, [Sigma-Aldrich]). At DIV5 and DIV11, 50% (1 ML) of the maintenance medium was replaced containing a vector, 200 µm of AICAR or 20 µm of H_2_O_2_, to reach a final concentration of resp. 100 µm and 10 µm.

### 4.8. In Situ Hybridization

Mouse brains were immediately snap-frozen using dry ice. In situ hybridization was performed as previously described (Ahd2 [49]⁠; Th [50]⁠).

Antibody list:
**Antibody****Product Number****Concentration**rabbit-α-H3K79me2Abcam, Ab35941:1000 in THZTrabbit-α-H3K79me3Abcam, Ab26211:750 in THZTRabbit-α-H3K27me3Millipore, 17–6221:1000 in THZTsheep-α-THMillipore, Ab15421:500 in THZTGoat-α-rabbit-555Life Technologies, A270391:1000 in PBSdonkey-α-sheep-488Life Technologies, A110151:500 in TBSchicken-α-GFPChicken-anti-GFP1:1000 in THZTrabbit-α-H3K79me1Abcam, ab28861:100 in THZTGoat-α-chicken-488Thermo Fisher, A-110391:1000 in PBSDonkey-α-rabbit-594Thermo Fisher, A-212071:1000 in PBS

### 4.9. Fluorescence Associated Cell Sorting (FACS)

Dissected midbrains were dissociated using a Papain (Worthington) and neurons were sorted using a BD FACS Aria III cell sorter. Gates were set on forward scatter versus side scatter, on forward scatter versus pulse width, and on forward scatter versus fluorescence channel 1 (528/38 filter; GFP fluorescence: sufficient to sort YFP positive neurons). Cells were collected in Trizol LS reagent (Invitrogen) ratio 1:3 in a precooled collection chamber at 4 °C and stored at −80 °C.

### 4.10. RNA Sample Generation

RNA from FACS isolated cells was purified using a Trizol LS (Sorted neurons, Invitrogen) according to the manufacturers protocol (3 volumes of PBS/cells:1 volume Trizol LS). For adult animals, brains were snap-frozen on dry ice, sliced in a cryostate until the anatomical markers that indicate the rostro-diencephalic DA area. Subsequently, 15 slices of 100 µm were sliced, the DA area was dissected, thawed, and lysed directly in 0.5 mL TriZOL (Invitrogen) and stored at −80 °C until further use. Three samples were pooled (using only one paired set of +/+, fl/+, and fl/fl from each nest/uterus) that were subsequently cleaned and DNAse treated using the Zymo Clean & Concentrator kit (Zymo Research). For primary neurons, 0.5 mL per 12 wells well was used.

### 4.11. RNA Sequencing

The quality of the RNA samples was determined using a fragment analyzer, samples with an RQN score > 6 passed QC. The NEBNext Ultra Directional RNA Library Prep Kit for Illumina was used to process the samples according to the protocol NEB #E7420S/L (New England Biolabs) to fragments of 300–500 bps. Clustering and DNA sequencing was performed using the Illumina NextSeq 500 device according to manufacturer’s protocols and with a minimum depth of 18 million quality-filtered reads per sample. The read counts were mapped against Mus musculus GRCm38.p4 and analyzed using DESeq2 to determine differential expression between groups (Genomescan BV).

### 4.12. Quantitative PCR

Total RNA was purified by applying Trizol (Invitrogen) to cells according to the manufacturer’s instructions. qPCR amplification was performed on a Roche light cycler using OneStep qPCR SYBR green kits (Qiagen, Hilden, Germany), according to the manufacturer’s protocol. A total of 10 ng RNA was used as the input.

List of Primers Used:
**Target****Forward Primer****Reversed Primer**ThTGCACACAGTACATCCGTCATGCGCAAATGTGCGGTACGCCAACAVmat2CCTCTTACGACCTTGCTGAAGGGCTGCCACTTTCGGGAACACATDatGGTGCTGATTGCCTTCTCCAGTGACAACGAAGCCAGAGGAGAAGPitx3CCTTCCAGAGGAATCGCTACCTCTGCGAAGCCACCTTGCACARspo2CCGAGCCCCAGATATGAACAGACCAACTTCACAACCTTCTACACalb2TGACTGCATCCCAGTTCCTGCTTGGACATCATGCCAGAACCCckTAGCGCGATACATCCAGCAGGTGGTATTCGTAGTCCTCGGCACTGirk2GGAACTGGAGATTGTGGTCATCCTCTTCCAGCGTTAGGACAGCTTCCAGAGTGAhd2GGAATACCGTGGTTGTCAAGCCCCAGGGACAATGTTTACCACGCNurr1CCGCCGAAATCGTTGTCAGTACTTCGGCTTCGAGGGTAAACGACTBPGAGAATAAGAGAGCCACGCTCACATCACAGCTCCCCACDot1lCTGGTGGCCCAGATGATTGAGTCCATGGTCTCTGCGTACT

### 4.13. Statistics and Graphs

For one-way analyses of the distribution of multiple [4] groups of nuclear H3K79 staining intensity in Figure 1C (age comparison), we have used a Kruskal–Wallis non-parametric test https://www.socscistatistics.com/tests/kruskal/default.aspx. To test if the distribution between two non-parametric groups differed Figure 1C (VTA/SNc comparison) we used Mann–Whitney analyses: https://www.socscistatistics.com/tests/mannwhitney/default2.aspx, followed by a Bonferroni correction for multiple testing. For data plotting, we have used in Figure 1, Figure 3 and Figure 4: https://huygens.science.uva.nl/PlotsOfData, by Dr. Joachim Goedhart. (Last accessed dates: 24 November 2022). To analyze the RNA sequencing data for differential expression, DESeq2 statistical analyses was used. For other statistics Student’s *t*-tests, with parameters as stated in the legends, were used.

## Data Availability

The RNAseq data is available under GSE184901 and GSE185257.

## Supplementary Materials

The following supporting information can be downloaded at: https://www.mdpi.com/article/10.3390/ijms24021387/s1.
