# Supplementary material for: Neuronal Dot1l Activity Acts as a Mitochondrial Gene-Repressor Associated with Human Brain Aging via H3K79 Hypermethylation
Source: Int J Mol Sci. 2023 Jan 10;24(2):1387. doi: 10.3390/ijms24021387 (PMC9862808; doi:10.3390/ijms24021387)
Supplement: Supplementary file 1 [file ijms-24-01387-s001.zip › ijms-2088845-supplementary.pdf]

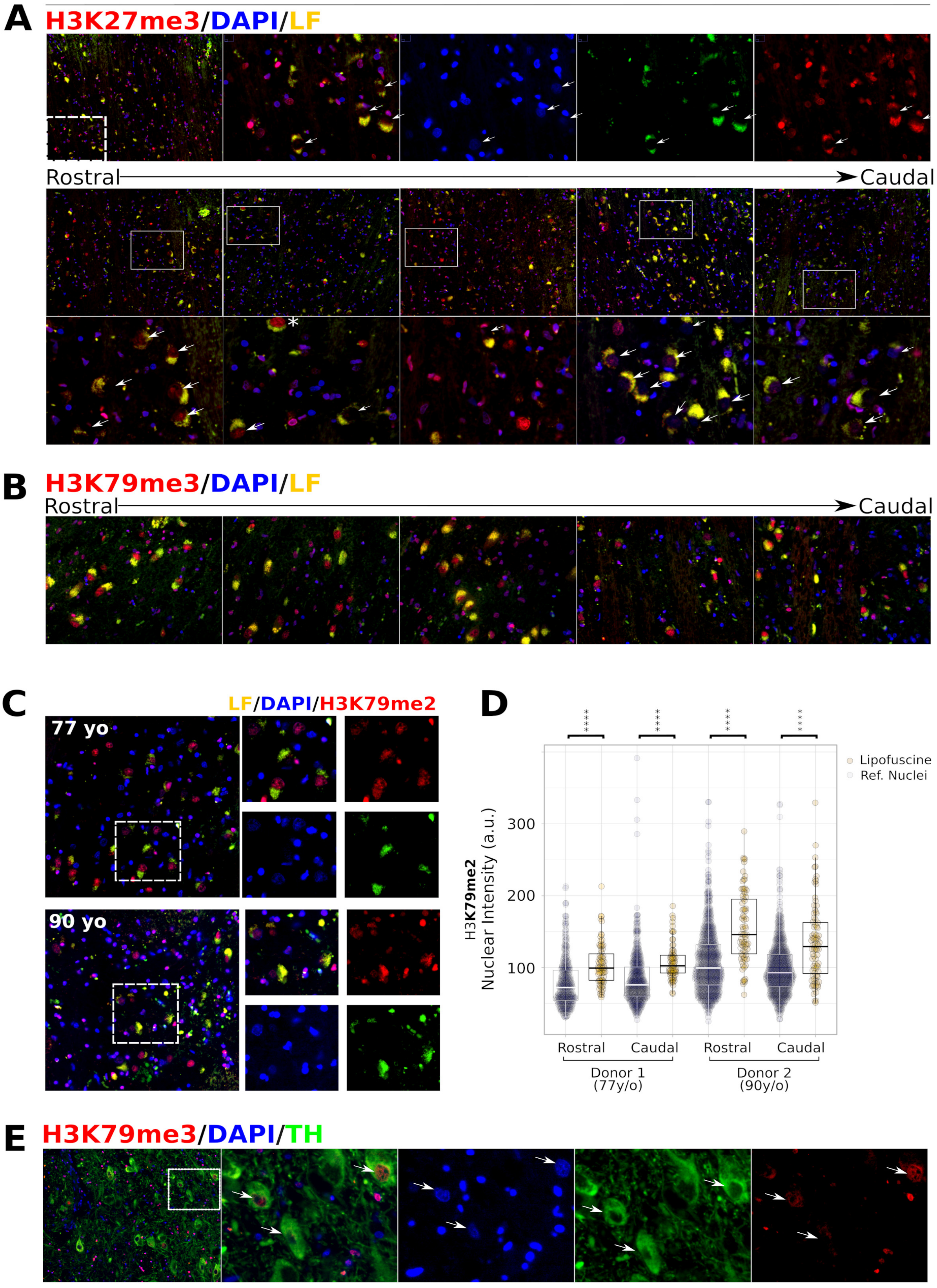

Supplemental Figure S1. Correlation between lipofuscin and H3K79 hypermethylation.

Rostral to caudal fluorescent microscopy images additional to Figure 1, of LF autofluorescence associated to large nuclei in the human midbrain of a 79 y/o male donor showing ER lysosomal/lipofuscin structures surrounding the nuclei relating to H3K27me3 (**A**) or H3K79me3 (**B**) staining with white arrows indicating large nuclei with low to almost absent H3K27me3 staining. (**C**) Fluorescent microscopy images of LF autofluorescence associated to large nuclei in the human midbrain of a 77 and 90 y/o. For the latter the white box depicted a few exceptions of relatively low H3K79me2 nuclear levels in the mids of LF associated nuclei with H3K79me2 hypermethylation. In (**D**), quantified levels of nuclear H3K79me2 are presented, comparing between nuclei that are surrounded by LF (yellow dots) and surrounding reference nuclei (blue dots). (**E**) Th (green), DAPI and H3K79me3 staining in 90-year-old asymptomatic control shows low to moderate H3K79me3 in aged Th+ SNc DA neurons. Statistics: Mann-Whitney test was performed for each separate section to test if H3k79me2 was increased in lipofuscine compared to reference nuclei. **** represents a *p*-value < 0.0001.

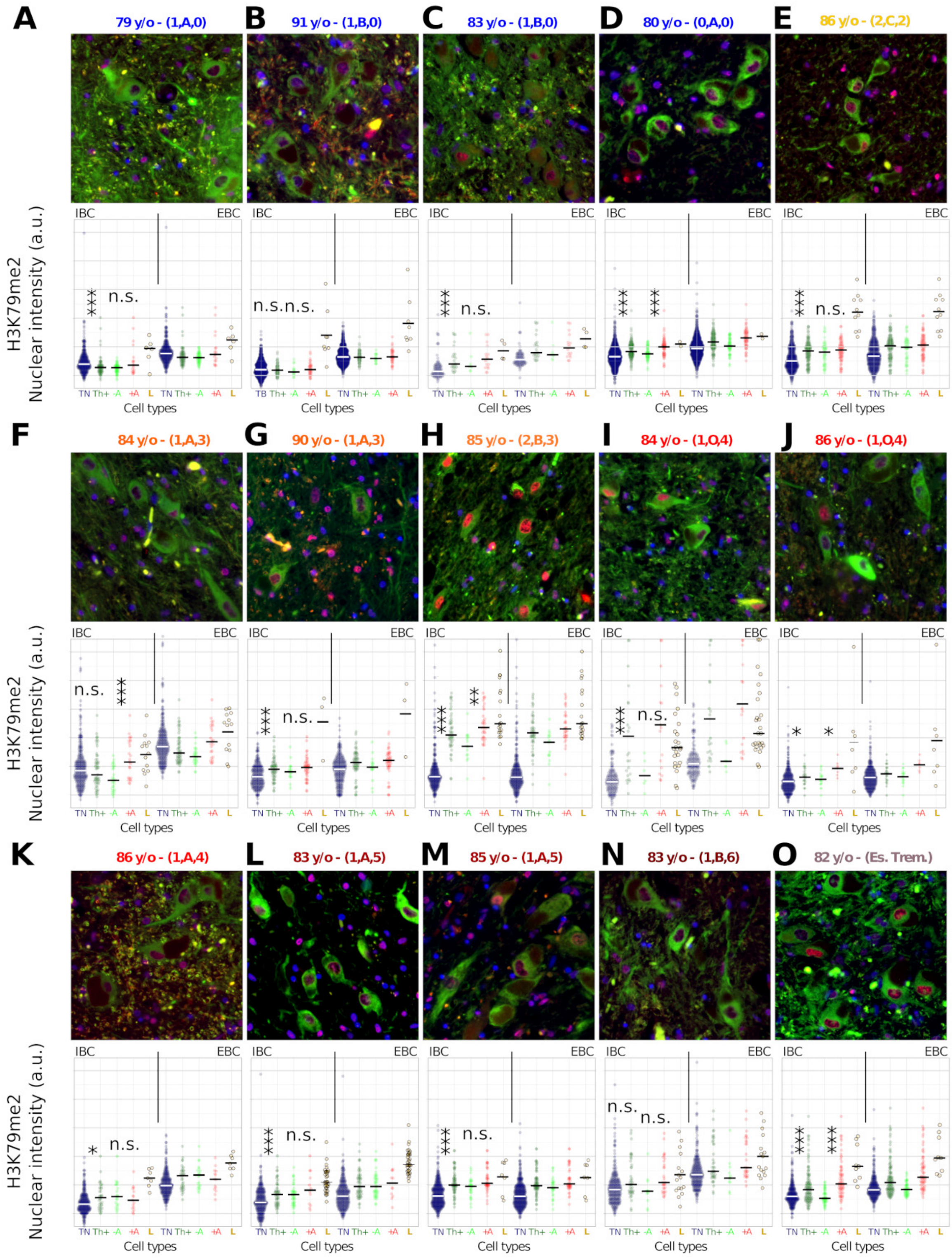

Supplemental Figure S2. Overview of single nucleus H3K79me2 levels between individuals and cell types.

For each donor (**A-O**), we have represented an image of TH+ neurons representing average levels of H3K79me2 staining intensity (a.u.) (upper panel) and each measure point (lower panel). The title colors and ages correspond to different Braak stages (which did not relate to K79me) levels. To visualize the independence of relative intra sample differences to local background, we have shown samples corrected for the local background, while the left panel are uncorrected data corrected for an individual background correction (IBC, described in 'Methods-quantification') on the left and corrected with an equal (no) background correction for all samples (EBC) on the right. From left to right, levels corresponding to non-DA nuclei (TH-negative (TN), blue), TH+ nuclei (TH+, dark green), and TH+ nuclei split into nuclei without clear pigmented inclusions (A-, light green) or with clear pigmented inclusions (red, +A). Lipofuscin positive neurons (LFn), were added as 5th condition for each subgraph (Yellow, L). Statistics: Performed on IBC measurements. Mann-Whitney analyses comparing between TH- and TH+ nuclei and between pigmented inclusions and lowly pigmented TH+ neurons adjusted with a post-hoc Bonferroni correction for multiple testing. **** represent $p < 0.0001$, *** $p < 0.001$, ** $p < 0.01$, * $p < 0.05$ and n.s.: non-significant.

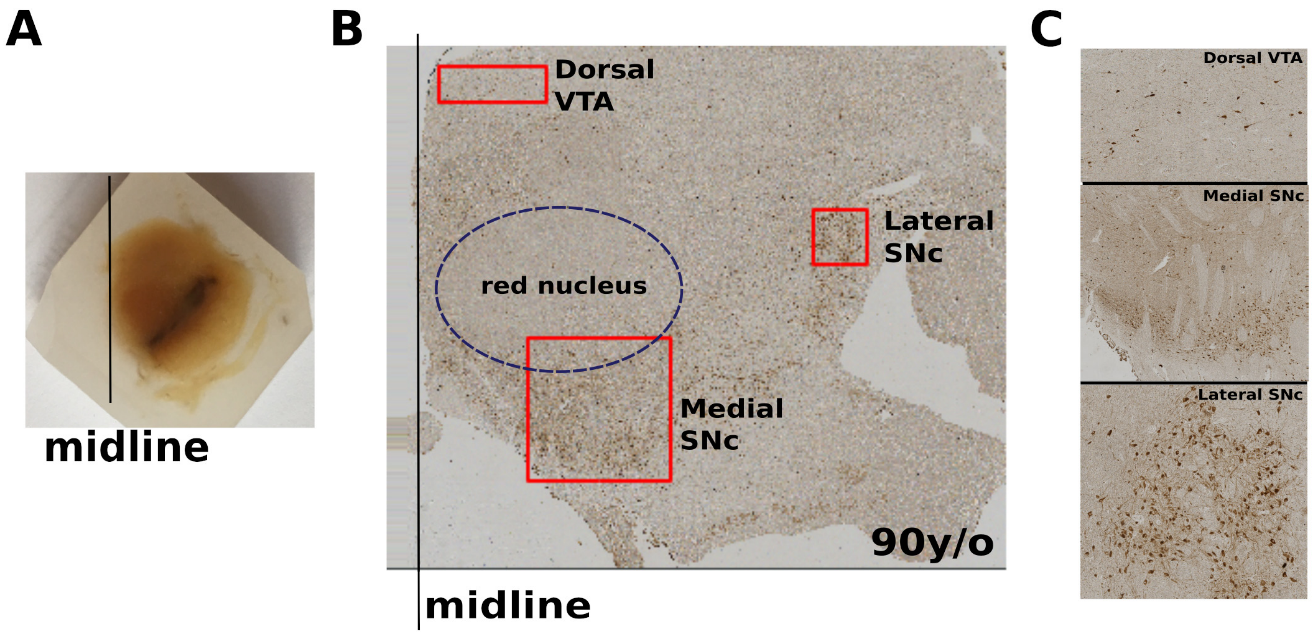

Supplemental Figure S3. Human midbrain material overview
(**A**) Example of paraffin donor material supplied by the Netherlands Brain Bank with (**B**) a coronal section stained with anti-TH DAB of a 90 year old donor. (**C**) Magnifications of TH+ cells in the dorsal VTA, the medial and lateral SNc. Blue dashed circle annotates the Red Nucleus; Red squares are regions that are highlighted in (**C**)

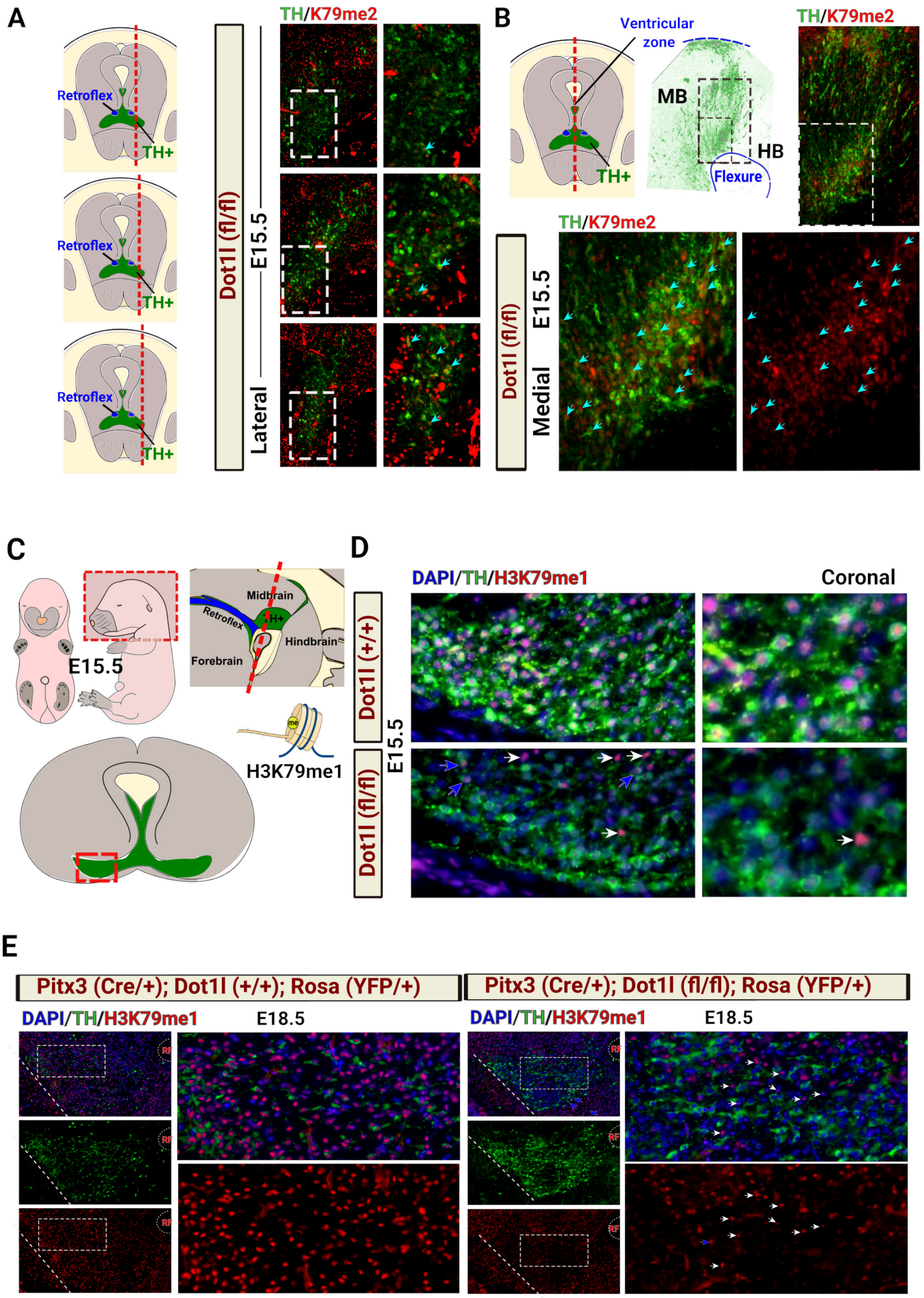

Supplemental Figure S4. Loss of H3K79 mono and dimethylation in post-mitotic dopamine neurons after ablation of Dot1l. (**A**) Sections increasingly lateral from the retroflex and (**B**) the most medial section of the E15.5 midbrain were studied to further investigate the loss of H3K79me2 following Dot1l depletion in dopamine neurons. In green Tyrosine Hydroxylase positive (TH+) cells were marked together with 1 staining nuclei H3K79me2 (red). Blue arrows indicate several Th+ neurons that still possess clear H3K79me2 nuclear staining, especially increasing when getting further lateral from the retroflex (**A**) and in the most medial sections of the midbrain (**B**) Blue arrows indicate two slightly H3K79me2 positive nuclei of TH+ cells. In (**C**) is a schematic overview of a E15.5 coronal midbrain section represented. The red dotted line represents the cutting position. The red box represents the region shown in (**D**). In (**D**) the loss of H3K79me1 is shown, supporting a global loss of H3K79 methylation in cKO Dot1l mice (lower panel), compared to controls (upper panel; Pitx3(Cre/+)). TH is shown in green, DAPI in blue and H3K79me1 in red. The white arrows indicate internal controls of non TH+ cells still clearly positive for H3K79me2. (**E**) Coronal sections of the left ventral midbrain of E18.5 Pitx3(Cre/+);Dot1l(+/+); Rosa26(YFP/+) (left half) and Pitx3(Cre/+);Dot1l(fl/fl);Rosa26(YFP/+) (right half) showing the absence of H3K79me1 (red) in nuclei (blue) of DA neurons (green; TH+) several days after the primary loss has been observed, suggesting Dot1l to be the sole factor to maintain global H3K79 methylation. White arrows indicate TH-negative cells within the SNc that possess normal H3K79me2 nuclear staining as internal controls (Floxed cells in this region are TH+ without exceptions).

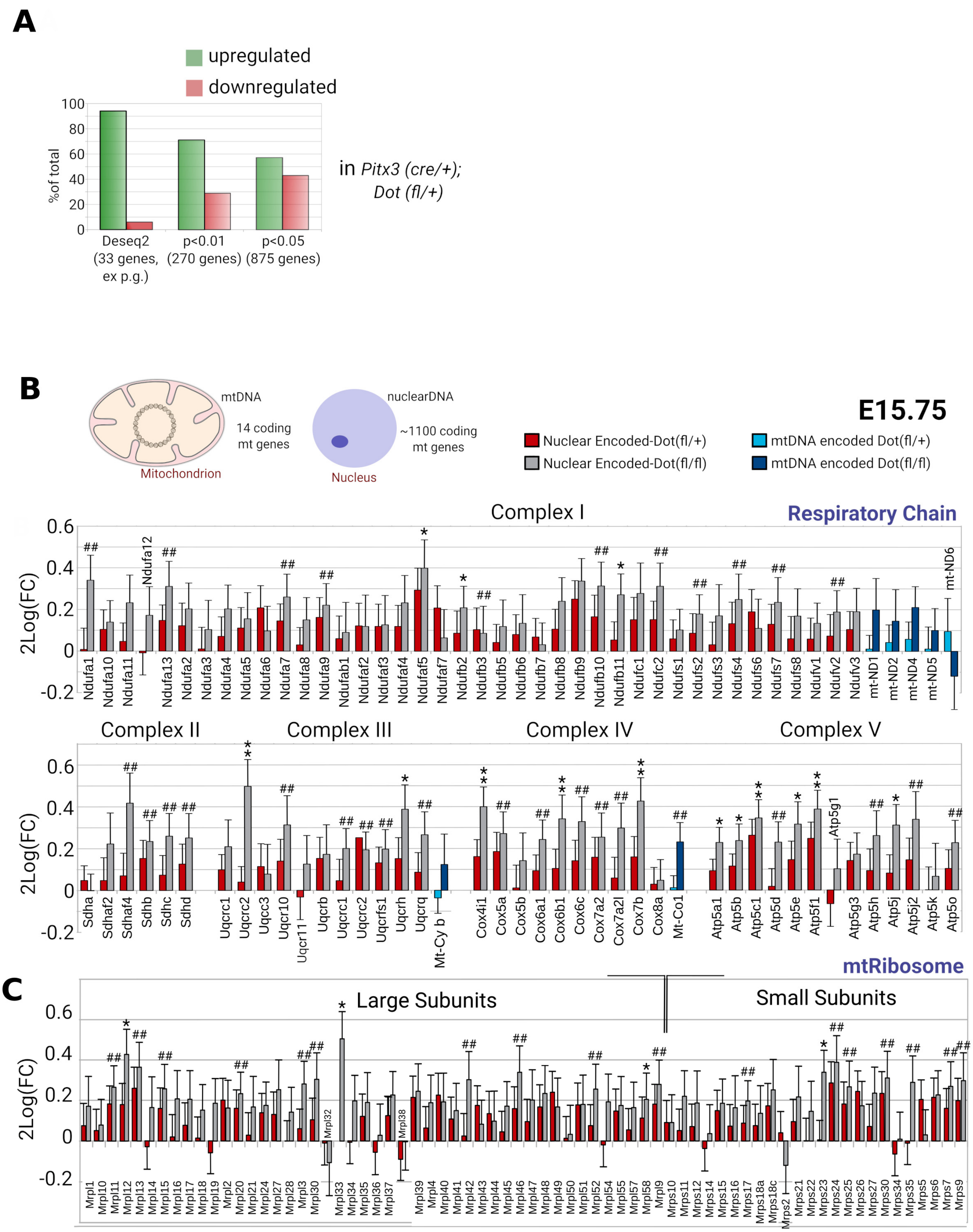

Supplemental Figure S5. Dot1l top deregulated genes are upregulated at 6 months and overlap with deregulated pseudogenes

(**A**) List of functions related to pseudogene parent genes to pseudogenes found deregulated with Deseq2 analyses of 6 months old Pitx3(Cre/+);Dot1l(fl/+) mice. (**B**) Number of genes up/down-regulated with different statistical cut-offs of 6 months old Pitx3(Cre/+);Dot1l(fl/+) mice transcriptomics data. From left to right, Deseq2 adjusted *p*-values < 0.05, or non-adjusted *p*-values (Wald-tests) > 0.01 and <0.05. (**C**) All retrieved respiratory chain genes at E15.75 sorted neurons. For nuclear-encoded genes, red bars represent (Dot1l(fl/+), gray bars represent Dot1l(fl/fl), and for mtDNA encoded genes: light blue bars represent (Dot1l(fl/+), dark blue bars represent Dot1l(fl/fl). (**D**) All retrieved mtRibosome encoding genes, red bars represent Dot1l(fl/+), gray bars represent Dot1l(fl/fl). Statistics: ** represent DEseq2, $p < 0.01$ and * $p < 0.05$; ## represent non-adj. $p < 0.05$ (Wald-test).

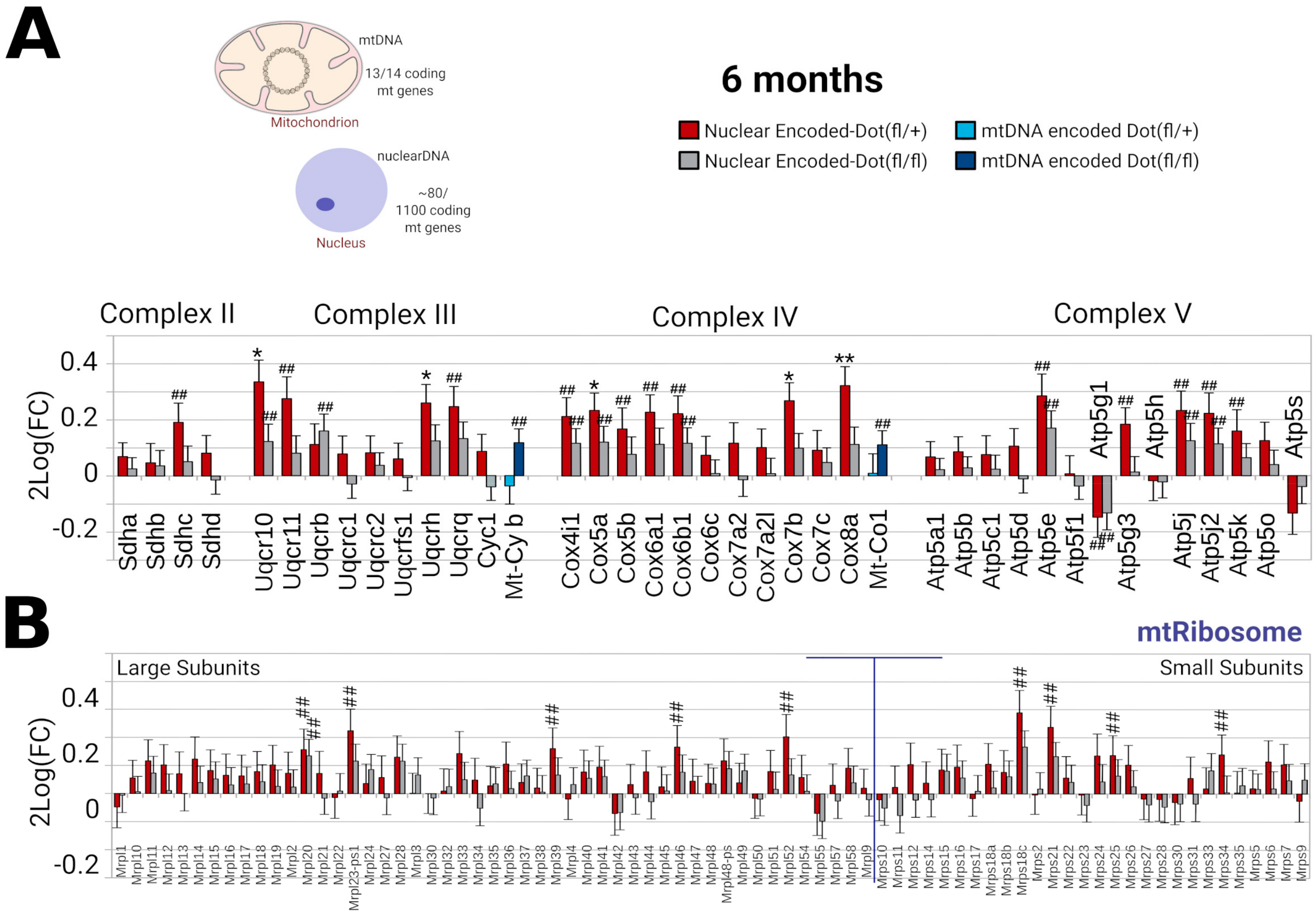

Supplemental Figure S6. Overview of respiratory genes regulated by Dot1l.

(**A**) Respiratory chain complex II-V retrieved from 6 months old Pitx3(Cre/+) induced dissected mouse midbrain transcriptomic profiles. For nuclear-encoded genes, red bars represent (Dot1l(fl/+), gray bars represent Dot1l(fl/fl), and for mtDNA encoded genes: light blue bars represent (Dot1l(fl/+), dark blue bars represent Dot1l(fl/fl). (**B**) mtRibosome encoding genes retrieved from 6 months old Pitx3(Cre/+) dissected mouse midbrain transcriptomic profiles, red bars represent Dot1l(fl/+), gray bars represent Dot1l(fl/fl). Statistics: ** represent DEseq2, $p < 0.01$ and * $p < 0.05$; ## represent non-adj. $p < 0.05$ (Wald-test).

Supplemental Table S1. Human donor specifications of midbrain material for 'Experiment 1'.

Age	Time of Death	Gender	PMD (h:min)	pH csf	AD & PD (Tgls, bA, aS)	Region	Cause of Death
45		M	8:50	6.71	0, 0, 0,	R1-R2	
49		M	6:15	6.26	0, 0, 0,	R1-R2	
77	09:35	M	8:25	7.19	1, 0, 0,	R1-R2-C1	CoD: Perforation of Bladder
90	10:20	F	6:05	6.12	3, B, 0	R1-R2-C1	CoD: Possible infection, Fever unknown foc.
79	7:40	F	7:40	6.02	1, A, 0	R2	CoD: Bronchopneumonia & Sepsis

Supplemental Table S2. Human donor specifications of midbrain material for 'Experiment 2'.

Age	Time of Death	Gender	PMD (h:min)	pH csf	AD & PD (Tgls, bA, aS)	Region	Cause of Death
79	20:35	M	5:20	6.72	1, A, 0	R1	CoD: Cachexia
91	13:20	F	4:15	6.5	1, B, 0	R2	CoD: Heart infarction
83	22:20	F	4:40	6.04	1, B, 0	R2	CoD: Ileus with pancreatic cancer
80	01:30	M	6:30	6.43	0, A, 0	R2	CoD: Ventricular fibrillation
86	21:00	F	6:25	6.39	2, C, 2	R2	CoD: not known; Diabetics, Breast Cancer, Renal Failure
84	16:50	M	4:50	6.41	1, A, 3	R2	CoD: Cachexia/End stage PD
90	14:25	F	4:50	6.37	1, B, 3	C1	CoD: Heart Rhythm, Resp. Tract Inf.
85	10:45	M	6:20	6.3	2, B, 3	R2	CoD: Cardic Arrest
84	02:40	M	6:30	6.4	1, 0, 4	R2	CoD: Myocard. Inf. Heart fibr.
86	02:25	M	5:35	6.19	1, 0, 4	R2	CoD: Aspiration Pneumonia
86	14:45	F	3:00	6.97	1, A, 4	R2	CoD: Seizure by Midazol. & Morf.
83	23:15	M	6:20	6.71	1, A, 5	R2	CoD: CVA (stroke)
85	06:59	M	8:31	6.49	1, A, 5	R1	CoD: Pneumonia & Heart failure
83	01:30	M	5:15	6.66	1, B, 6	R1	CoD: Pneumonia
82	15:07	F	7:08	6.28	1, A, 0	R1	CoD: Dehydration
